# Photonic waveguide chip–based nanoscopy visualizes rearrangements of the cortical actin cytoskeleton in activated Jurkat T cells

**DOI:** 10.1126/sciadv.aeg3960

**Published:** 2026-07-15

**Authors:** Surjendu Bikash Dutta, Wolfgang Hübner, Paul Goffing, Anders Kokkvoll Engdahl, Stefan Belle, Ralf Hellmann, Mark Schüttpelz, Francesco Dell’Olio, Thomas Huser

**Affiliations:** ^1^ Biomolecular Photonics, Faculty of Physics, University of Bielefeld, Bielefeld, Germany.; ^2^ Applied Laser and Photonics Group, Aschaffenburg University of Applied Sciences, Aschaffenburg, Germany.; ^3^ Micro Nano Sensor Group, Polytechnic University of Bari, Bari, Italy.

## Abstract

The actin cytoskeleton in activated T cells undergoes rapid structural changes during the formation of an immunological synapse. Superresolution fluorescence microscopy provides excellent means to visualize such antibody-triggered changes. Here, we use single-molecule localization microscopy (SMLM) enabled by transparent polymer waveguide chips to resolve the filamentous-actin (F-actin) cytoskeleton in activated Jurkat T cells in comparison to nonactivated T cells across a large field of view. Transparent polymer waveguides enable a wide array of imaging modalities. In combination, these modalities reveal the structural differences between lamellipodial and ramified actin networks within the immunological synapse of activated T cells. SMLM images recorded by using narrow-width waveguide total internal reflection illumination resolve the double-stranded helical structure of actin filaments in activated Jurkat T cells. The average crossover length of the filaments is measured to be ~40 nanometers, which corroborates similar observations of isolated actin filaments by electron microscopy.

## Teaser

Superresolution optical fluorescence imaging reveals that immune cells adapt their scaffold structures on the nanoscale.

## INTRODUCTION

T lymphocytes play an important role in the adaptive immune response and usually undergo a series of shape changes and signaling steps when encountering antigen-presenting target cells in the body (*1*–*3*). The contact and binding of T cell receptors (TCRs) with antigens on the surface of antigen-presenting target cells result in the formation of TCR microclusters and lead to the reorganization of the cell surface and the formation of an immunological synapse (IS) (*2*, *4*, *5*). Cortical actin is a key cellular component that involves various major cell functions such as regulating cell morphology, cell division, T cell activation, etc. (*3*, *6*–*8*). Filamentous actin networks influence signaling, TCR microcluster trafficking, and cytoskeleton rearrangements at the IS (*1*–*3*, *5*–*7*, *9*–*11*). Therefore, imaging and quantitative assessment of cortical actin networks are essential to describe the important biological processes associated with immune cells (*3*, *9*). Electron microscopy (EM) has mostly been deployed in the past to image and understand the structure of the T cell cytoskeleton. However, the overall conditions, especially the exposure to a high-energy electron beam, limit the use of EM in investigating the complex organization of the actin network in T cells (*9*, *12*).

During the past two decades, superresolution fluorescence microscopy (SRM), often also designated as optical nanoscopy, has shown immense potential for visualizing subcellular structural changes and understanding cellular processes and interactions beyond the diffraction limit of light (*13*–*17*). Several SRM techniques, such as structured illumination microscopy (SIM) (*18*, *19*), stimulated emission depletion microscopy (STED) (*20*, *21*), single-molecule localization microscopy (SMLM) (*22*, *23*), and temporal and spatial signal fluctuation–based techniques (superresolution optical fluctuation imaging, entropy-based superresolution imaging, superresolution radial fluctuations, and enhanced superresolution radial fluctuations) (*24*–*27*), have demonstrated their potential for such applications in a large body of publications. Optical nanoscopy has provided key advantages for imaging the cellular ultrastructure, e.g., by being relatively noninvasive, providing more specific contrast, and enabling the simultaneous imaging of multiple fluorescently labeled species (*13*, *17*, *28*, *29*). Despite all of these benefits, the complex rearrangement of the actin cytoskeleton and the dynamics of IS formation during T cell activation with subdiffraction resolution have yet to be unraveled. The actin cortex mostly supports the cell membrane and is composed of polydisperse filaments with an extremely high density at the IS periphery, i.e., the basal lamellipodium (*3*, *6*). SMLM techniques allow us to assess the distribution of actin filaments at T cell synapses with subnanometer precision (*30*). SIM using total internal reflection (TIRF) illumination, on the other hand, can assess cortical actin flow and actin dynamics at each stage of the T cell activation process with high temporal and moderate spatial resolution but is currently inadequate for disclosing the distinct individual filamentous structure of the dense lamellipodial meshwork as well as the ramified actin network below the IS (*6*, *11*).

Several recent studies based on SRM have recently reported the observation and a better understanding of the organization of cortical actin networks and their dynamics following T cell activation (*9*, *11*, *31*). For example, Ashdown *et al.* (*11*) used TIRF-SIM and spatiotemporal image correlation spectroscopy to image filamentous-actin (F-actin) and plasma membrane dynamics at the T cell synapse. Fritzsche *et al.* (*3*) resolved the architecture of the ramifying actin network underpinning the IS and discussed the global actin cytoskeleton rearrangements in resting and surface-activated T cells using STED microscopy. Peters *et al.* (*32*) used image reconstruction by integrating exchangeable single-molecule localization to dissect the architecture of cortical actin at the T cell IS. Yi *et al.* (*33*) used the multiplexed direct stochastic optical reconstruction microscopy (dSTORM) technique (madSTORM) to probe individual molecules that are involved in the TCR signaling cascade near the activated surface of a T cell. Gustafsson *et al.* (*26*) applied algorithmic superresolution radial fluctuations to image LifeAct-GFP (green fluorescent protein)–expressing actin filaments in Jurkat T cells. Although these SRM techniques try to demonstrate the complex organization of actin filaments in T cell synapses, all encounter challenges with regard to the characterization of the ramified actin network and the identification of its individual filamentous structure with subnanometer spatial resolution within the dense meshwork, and they provide rather low throughput. Therefore, a superresolution optical microscopy technique that enables us to resolve the cytoskeletal structure in immune cells with high throughput and high spatial resolution is preferred, which could then also be used to reveal the complex architecture of the dense cortical actin network in T cells at the IS.

Optical nanoscopy based on photonic waveguide chip–enabled TIRF fluorescence (WG-TIRF) excitation has demonstrated several advantages over conventional objective-type TIRF microscopy (*34*–*38*) in recent years. In conventional objective-type TIRF microscopy, the excitation and emission paths are shared for epi-illumination and fluorescence detection, which restricts this modality to high–numerical aperture (NA) lenses with a limited field of view (FOV). In WG-TIRF nanoscopy, however, an evanescent field (EF) is generated on top of the waveguide surface (often consisting of high-NA materials) across a large and flat surface area of the planar waveguide. In addition, the photonic waveguide chips serve as sample holders to conveniently move and register the sample between different imaging setups (*39*). Thus, waveguide chips have become a versatile platform to perform multimodal optical nanoscopy over a large FOV with subnanometer spatial resolution (*34*–*36*, *39*). Several recent studies, including our own works, have demonstrated in detail the advantages of the use of transparent waveguide chips for TIRF nanoscopy over waveguides fabricated on opaque substrates (*39*–*41*). Briefly, transparent photonic chips provide a universal imaging platform, where fluorescence can be collected through the substrate via an inverted microscope configuration. This still permits the use of high-resolution oil immersion objective lenses for fluorescence detection, which are usually aberration corrected for a 170-μm-thick cover glass substrate (*39*–*42*).

Here, we present the superresolution imaging of the rearrangement of cortical actin filaments at ISs in antibody-activated Jurkat T cells facilitated by a transparent polymer photonic waveguide chip. The use of transparent waveguides permits us to exploit and compare results of at least three different nanoscopy modalities with the same sample: waveguide TIRF–based nanoscopy, high-NA objective-type TIRF nanoscopy, and three-dimensional SIM (3D-SIM) nanoscopy. WG-TIRF excitation reduces the background signals and reveals the differences between lamellipodial and ramified actin networks at ISs of activated T cells depending on the EF distribution, generated upon close contact with the waveguide surface. We find that dSTORM images recorded using TIRF illumination in narrow-width waveguides were able to resolve the double-stranded helical structure of actin filaments in activated Jurkat T cells. Furthermore, the average crossover length of the filaments was measured to be ~40 nm, which was previously only seen by using EM. We compare these results with 3D-SIM imaging and objective-type TIRF illumination dSTORM imaging of actin filaments. This reveals that the structural design of transparent polymer waveguide chips and its resulting EF distribution not only enabled us to visualize the functional rearrangement of the dense cortical actin network in Jurkat T cells at the IS with high throughput (FOV of ~180 μm by 120 μm) and high spatial resolution (~15 nm), but it furthermore allowed us to image the differences between the actin network at the circumference of the IS and the ramified actin network in the center of the IS.

## RESULTS AND DISCUSSION

Analyzing the ultrastructural actin cytoskeleton arrangements in nonactivated and antibody-activated T cells with SRM unravels the complex role that actin networks play in IS formation (*3*). The primary focus of our study is the development of a simple optical method for imaging the actin cytoskeleton of T cells at specific time points during biological processes, such as cell-cell interactions, cell migration, etc., on the nanoscale level and with a large FOV. To visualize the actin cytoskeleton of T cells interacting with a substrate by superresolution fluorescence imaging, the Jurkat T cells were first seeded on top of poly-l-lysine (PLL)–coated microscope coverslips and then followed by fixation and fluorescence staining. Afterward, SMLM images of actin filaments in fixed Jurkat T cells labeled with Alexa Fluor 647 Phalloidin were recorded using dSTORM measurements. dSTORM allows us to identify the filaments’ structure with high spatial resolution by localizing individual fluorescent molecules with up to ~10- to 20-nm precision (*39*, *43*).

**Fig. 1.**
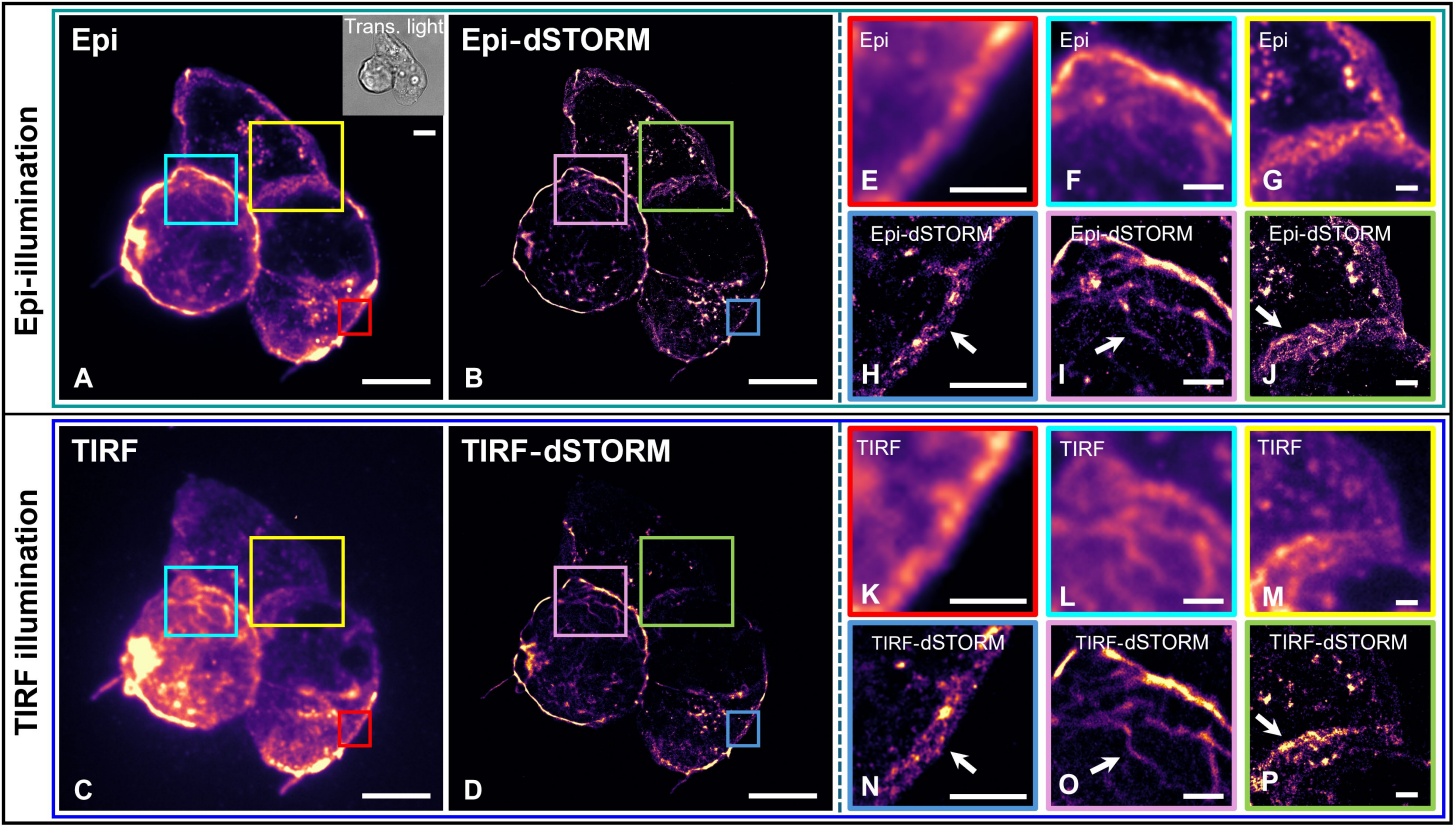
dSTORM images of actin filaments of fixed, nonactivated Jurkat T cells attached to the coverslip surfaces. (**A** and **B**) Epi-illumination diffraction-limited wide-field (Epi) and corresponding dSTORM (Epi-dSTORM) images of fixed Jurkat T cells, respectively. The gray scale inset shows the trans-illuminated bright-field image (Trans. light) of the same cells. (**E** to **J**) Enlarged view of Epi (E to G) and Epi-dSTORM (H to J) images of the cells marked with red, cyan, and yellow boxes and dark blue, plum, and light green boxes, respectively. (**C** and **D**) Objective-type TIRF excitation diffraction-limited (TIRF) and corresponding dSTORM (TIRF-dSTORM) images of the same fixed T cells, respectively. (**K** to **P**) Enlarged view of the TIRF (K to M) and TIRF-dSTORM (N to P) images of the cells marked with red, cyan, and yellow boxes and dark blue, plum, and light green boxes, respectively. Both Epi-dSTORM and TIRF-dSTORM measurements distinguish individual actin filaments with cross-sectional widths well below the diffraction limit. From the TIRF images, it appears that only the circumference of T cells is attached to the glass surface, and most of the remaining parts are not properly attached. The spatial resolutions of both Epi-dSTORM and TIRF-dSTORM images are determined by FRC analysis and are ~45 and ~40 nm, respectively. Actin filaments are stained with Alexa Fluor 647 Phalloidin. Scale bars, 5 μm (A to D) and 1 μm (E to P).

First, we imaged nonactivated T cells adhering to a PLL-coated microscope coverslip surface by epi-illumination and by objective-type TIRF illumination. For both modalities, we also acquired corresponding dSTORM images of the same region, as shown in Fig. 1. Please note that for better comparison, all images were taken at the same location and of the same cells [which are also shown in the trans-illuminated bright-field image (Trans. light) in the inset to Fig. 1A]. None of these images show a notable number of well-distinguishable actin filaments. While this is expected for epi-illuminated wide-field and dSTORM images [in Epi and Epi-dSTORM images in Fig. 1 (A and B) and enlarged regions of interest (ROIs) in Fig. 1 (E to J)], which average such information along the axial direction, the TIRF images also exhibit the same behavior [in TIRF and TIRF-dSTORM images in Fig. 1 (C and D) and ROIs in Fig. 1 (K to P)]. Although the Jurkat T cells appear to adhere well to the PLL-coated surface, it seems that only a small number of actin filaments are present at the edges of the immobilized cells, whereas most parts of the cells do not form distinguished actin-based structures such as lamellipodia, lamella, actin arcs, etc. Both Epi-dSTORM and TIRF-dSTORM measurements are able to distinguish a few individual actin filaments with cross-sectional widths well below the diffraction limit. The spatial resolutions for both dSTORM measurements are determined by Fourier ring correlation (FRC) (*44*) analysis to be ~45 and ~40 nm, respectively. This distinct absence of intricate actin networks could potentially be attributed to the shape of nonactivated Jurkat T cells, which remain mostly round and do not seem to form any kind of notable actin rearrangement on the glass surface. The images shown in Fig. 1 are representative of this situation, and we observed the same behavior by performing five similar experiments with T cells on PLL-coated coverslip surfaces.

**Fig. 2.**
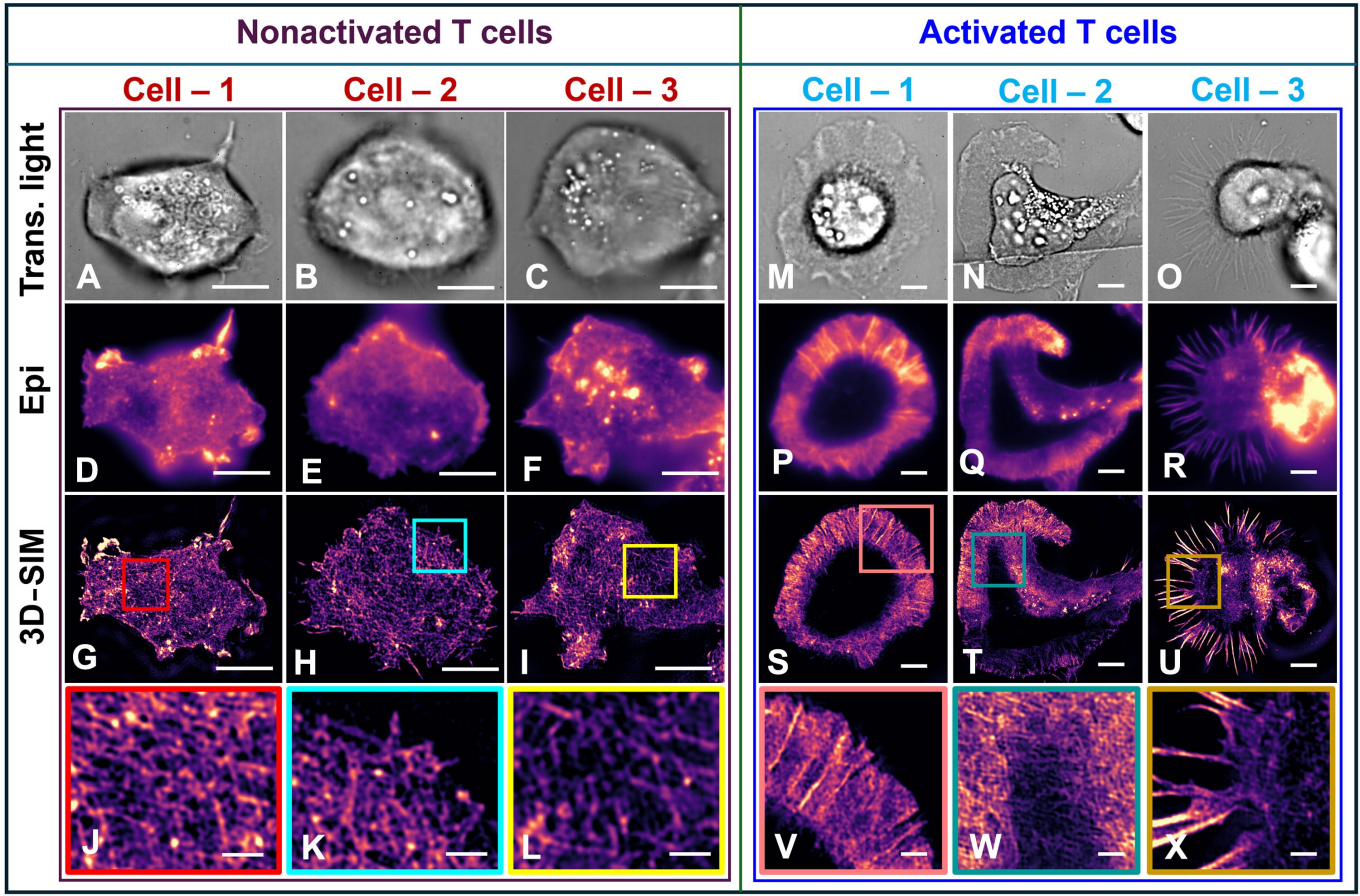
Diffraction-limited wide-field fluorescence (Epi) and superresolution 3D-SIM images of actin filaments in fixed Jurkat T cells attached to polymer waveguide surfaces. The left-hand side of the figure, separated by a green line, presents the actin filament structure of nonactivated T cells, which are attached to the FN-coated waveguide surfaces. The right-hand side of the figure shows the actin cytoskeleton structure of activated T cells attached to antibody-coated waveguide surfaces. (**A** to **F**) Trans-illuminated bright-field (Trans. light) (A to C) and diffraction-limited wide-field (Epi) (D to F) images of three different nonactivated T cells, respectively. (**G** to **I**) Corresponding reconstructed 3D-SIM images of filamentous actin structures of nonactivated fixed T cells attached on top of the FN-coated waveguide surface. (**J** to **L**) Enlarged views of the regions of interest marked with red, cyan, and yellow boxes, respectively. A substantially denser mesh of actin filaments can be seen spread all over the cell surface. (**M** to **R**) Trans. light (M to O) and Epi (P to R) images of three different surface-activated T cells, respectively. (**S** to **U**) Representative reconstructed 3D-SIM images of distinct filamentous structures of the cortical actin network in activated T cells. (**V** to **X**) Enlarged views of the region of interest marked with rose, dark teal, and orange boxes, respectively. Actin cytoskeleton rearrangements of three different Jurkat T cells after contact with activated (functionalized) waveguide surfaces. Cells were activated for an equal duration of time. Actin filaments are stained with Alexa Fluor 647 Phalloidin. Scale bars, 5 μm (A to I and M to U) and 1 μm (J to L and V to X).

To compare this behavior of T cells with that of T cells attached to the surface with other properties, we further seeded the cells on transparent polymer waveguides. Jurkat T cells were seeded on fibronectin (FN)–coated and antibody-activated transparent polymer photonic waveguide chips. Transparent planar polymer waveguides (EpoCore as the core, refractive index *n* = 1.59 for λ = 647 nm) of varying width and with a thickness of 1.2 μm were fabricated on top of a standard #1.5 borosilicate microscope coverslip substrate (*39*, *40*). The excitation laser beam propagates through the waveguide core material and generates an EF on top of the waveguide surface because of TIRF. The EF decays within a penetration depth of ~150 to 200 nm and is used to excite all fluorescent markers located within this small zone above the waveguide surface. The transparent nature of the polymer-based photonic waveguide chips enables both objective-type and waveguide-based TIRF excitation of the same region of the waveguide surface. Furthermore, it allows for the very efficient collection of fluorescence emissions with a high-NA objective lens. Jurkat T cells attached to FN-coated waveguides are not activated and referred to as nonactivated (or resting) T cells. Jurkat T cells attached to waveguide chips coated with immunoglobulin G (IgG) and anti-CD3 and anti-CD28 antibodies, however, are activated and undergo cytoskeletal changes upon IS formation. To quickly evaluate the actin cytoskeleton in nonactivated and activated T cells on top of polymer waveguide surfaces by superresolution microscopy, 3D-SIM measurements of cells on these substrates were carried out. Figure 2 shows 3D-SIM images of actin filaments in fixed Jurkat T cells attached to waveguide surfaces. The left panel of the figure separated by a light green line shows the actin network in three different nonactivated T cells. The right panel of the figure, on the other hand, shows the actin cytoskeleton of three different antibody-activated T cells. The trans-illuminated bright-field (Trans. light), diffraction-limited wide-field fluorescence (Epi), and corresponding reconstructed 3D-SIM fluorescence images of filamentous actin networks of nonactivated T cells are shown in Fig. 2 (A to I, respectively). The enlarged views of ROIs, marked with red, cyan, and yellow boxes, are presented separately in the same figure (Fig. 2, J to L). The 3D-SIM images (shown as maximum intensity projection) reveal a distinct and substantially denser mesh of filamentous actin structures in nonactivated T cells that spread across the entire cell surface. It is apparent that in nonactivated T cells, the cell cytoskeleton exhibits an isotropic distribution of cortical actin filaments, which do not provide the ability to function as a transportation network. Figure 2 (M to U), on the other hand, represents the Trans. light, Epi, and corresponding reconstructed 3D-SIM images of cortical actin networks of three different surface-activated Jurkat T cells, respectively. Enlarged views of the ROIs marked with rose, dark teal, and orange boxes are seen in Fig. 2 (V to X). Here, it is apparent that the T cells contacting the antibody-activated surface spread out and assemble in a dense cortical network, forming a rosette-shaped actin structure at the leading edge of the interface, resulting in the basal lamellipodium. 3D-SIM further reveals a highly ramified actin network in activated T cells slightly above the glass surface (see, e.g., Fig. 2W), which is connected to the lamellipodium where the cells are attached to the waveguide surface. In a later stage of activation or when the cells are fully activated, the ramified and lamellipodial networks contract and exhibit a complex dense structure of actin filaments radiating toward the center of the interface (see, e.g., Fig. 2X).

These results confirm that in contrast to the antibody-activated (or functionalized) surface, Jurkat T cells attached to the bare FN-coated surface are neither polarized nor do they exhibit a ramified network. Furthermore, the transparent polymer waveguide surface has no effect on cell adhesion, T cell activation, image acquisition, or the 3D-SIM image reconstruction. It is, however, also apparent that although the resolution gain of 3D-SIM allows us to image the cytoskeletal rearrangement of cortical actin with a spatial resolution of ~120 nm laterally and 300 nm axially, this is insufficient to resolve the very dense spatial organization of actin filaments at the basal membrane.

**Fig. 3.**
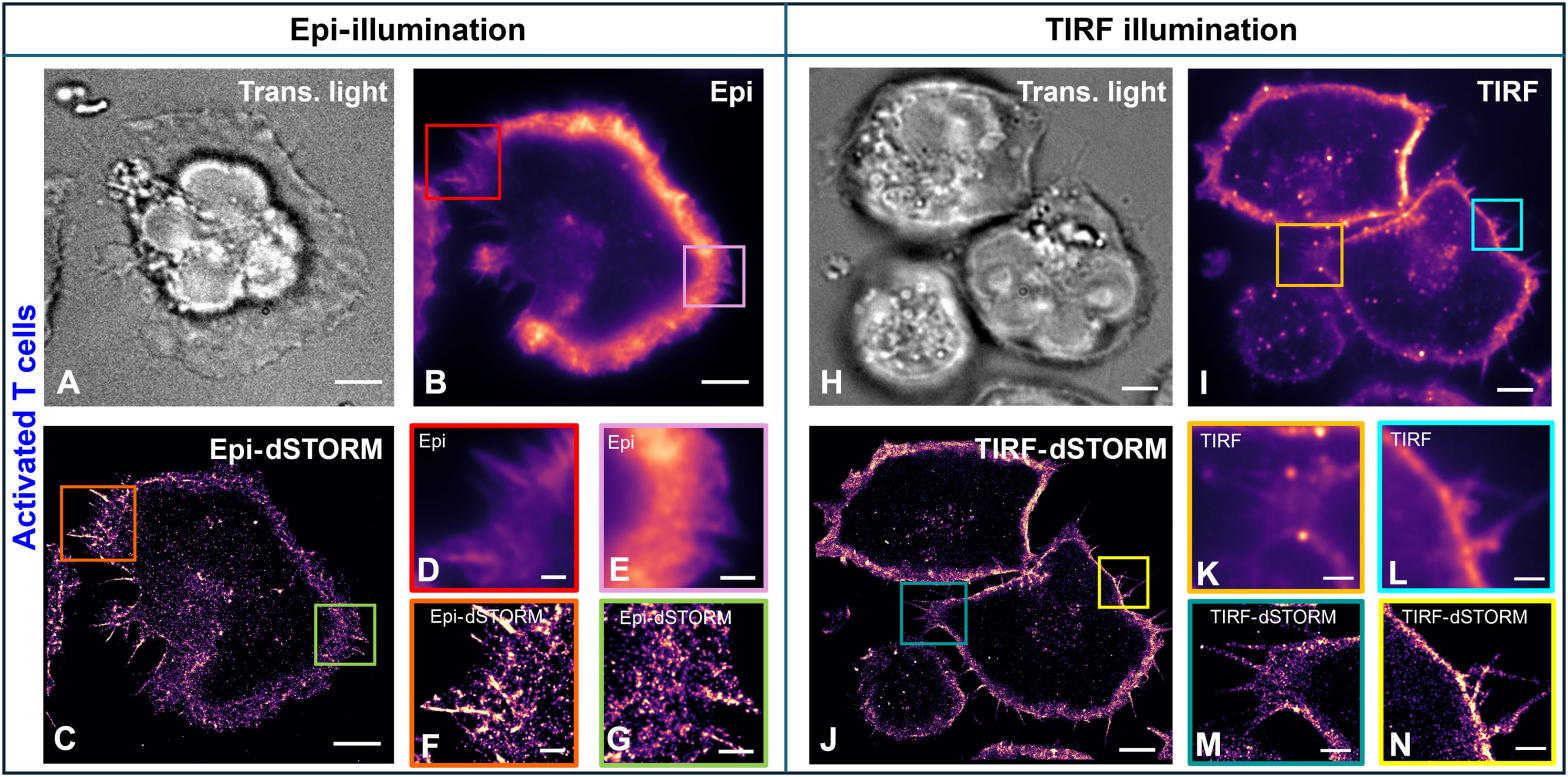
dSTORM images of actin cytoskeletal rearrangements in activated fixed Jurkat T cells. The cells shown in the two left-hand columns are excited with epi-illumination, and the cells shown in the two right-hand columns are excited with objective-type TIRF illumination. (**A** and **B**) Trans. light and Epi images of filamentous actin structures in activated T cells, respectively. (**C**) Corresponding Epi-dSTORM image of actin networks in the same activated T cells. (**D** to **G**) Representative enlarged views of regions of interest in Epi (marked in red and plum) (D and E) and Epi-dSTORM images (marked in orange and light green) (F and G) of actin filaments in activated T cells. (**H** and **I**) Trans. light and TIRF images of filamentous actin structures in activated T cells, respectively. (**J**) Corresponding TIRF-dSTORM image of the actin network in the same activated T cells. (**K** to **N**) Representative enlarged views of regions of interest in TIRF (marked in amber and cyan) (K and L) and TIRF-dSTORM (M and N) images (marked in dark teal and yellow) of actin filaments in activated T cells. Actin cytoskeletal rearrangements were observed in T cells following contact with the activating antibody-coated waveguide surface. Actin filaments are well resolved in both Epi-dSTORM and TIRF-dSTORM images. However, the peripherally forming lamellipodial structures comprised a highly dense network of filaments. Actin filaments are stained with Alexa Fluor 647 Phalloidin. Scale bars, 5 μm (A to C and H to J) and 1 μm (D to G and K to N).

Accordingly, we used Epi-dSTORM and TIRF-dSTORM to image the cortical actin network in activated Jurkat T cells at even higher spatial resolution (see Fig. 3). Figure 3 (A to C) presents the Trans. light, Epi, and corresponding Epi-dSTORM images of the lamellipodial actin network in activated T cells. Enlarged views of the ROIs marked in red and plum (Epi) and orange and light green (Epi-dSTORM) are also shown in the same figure (Fig. 3, D to G). Figure 3 (H to J) shows the Trans. light, TIRF, and corresponding TIRF-dSTORM images of actin filaments in activated T cells. Again, ROIs marked in amber and cyan (TIRF) and dark teal and yellow (TIRF-dSTORM) are also shown (Fig. 3, K to N). As expected, both Epi-dSTORM and TIRF-dSTORM images explicitly allow us to visualize individual actin filaments with improved spatial resolutions of ~35 nm and ~30 nm for Epi-dSTORM and TIRF-dSTORM imaging, respectively.

The results shown in Figs. 2 (M to X) and 3 reveal that activated T cells become polarized by decreasing their height, extending their leading edge, and spreading evenly across the antibody-coated surface. The dSTORM images suggest that upon contact with the antibody-coated surface, the dorsal ruffling stops and the T cells form a ring-like symmetric contact with the surface followed by the formation, undulation, and contraction of the peripheral lamellipodium. The peripherally formed ring-like lamellipodial structures are composed of a highly dense network of filaments, which was not fully resolvable by TIRF-dSTORM imaging, especially the densely organized short filaments. Furthermore, it appears that the dense F-actin structure is overlaid by another long actin filament network, which seems to be pointing toward the center of the cellular interface. The ramified actin network underneath the central region of the T cell IS, however, remained elusive for TIRF-dSTORM imaging. Therefore, we took advantage of the larger FOV and the penetration depth of the EF created by illumination through the transparent photonic waveguide chips, as shown in fig. S1 (Supplementary Materials). In general, a large part of the signal of single-molecule fluorescence will be directed toward the material with the higher index of refraction, in this case, the transparent polymer waveguides. However, given that the detection optics in the epi-detected modality is based on a microscope objective with index oil, a notable portion of this fluorescence is still being detected by the objective lens. Hence, before further measurements with waveguide TIRF illumination, we determined the single-molecule brightness values recorded for molecules on the bare coverslip surface and on the waveguide surface when both are excited by objective-type TIRF illumination and did not find any notable difference between them. The detailed measurements and analysis are presented in fig. S2 (Supplementary Materials).

**Fig. 4.**
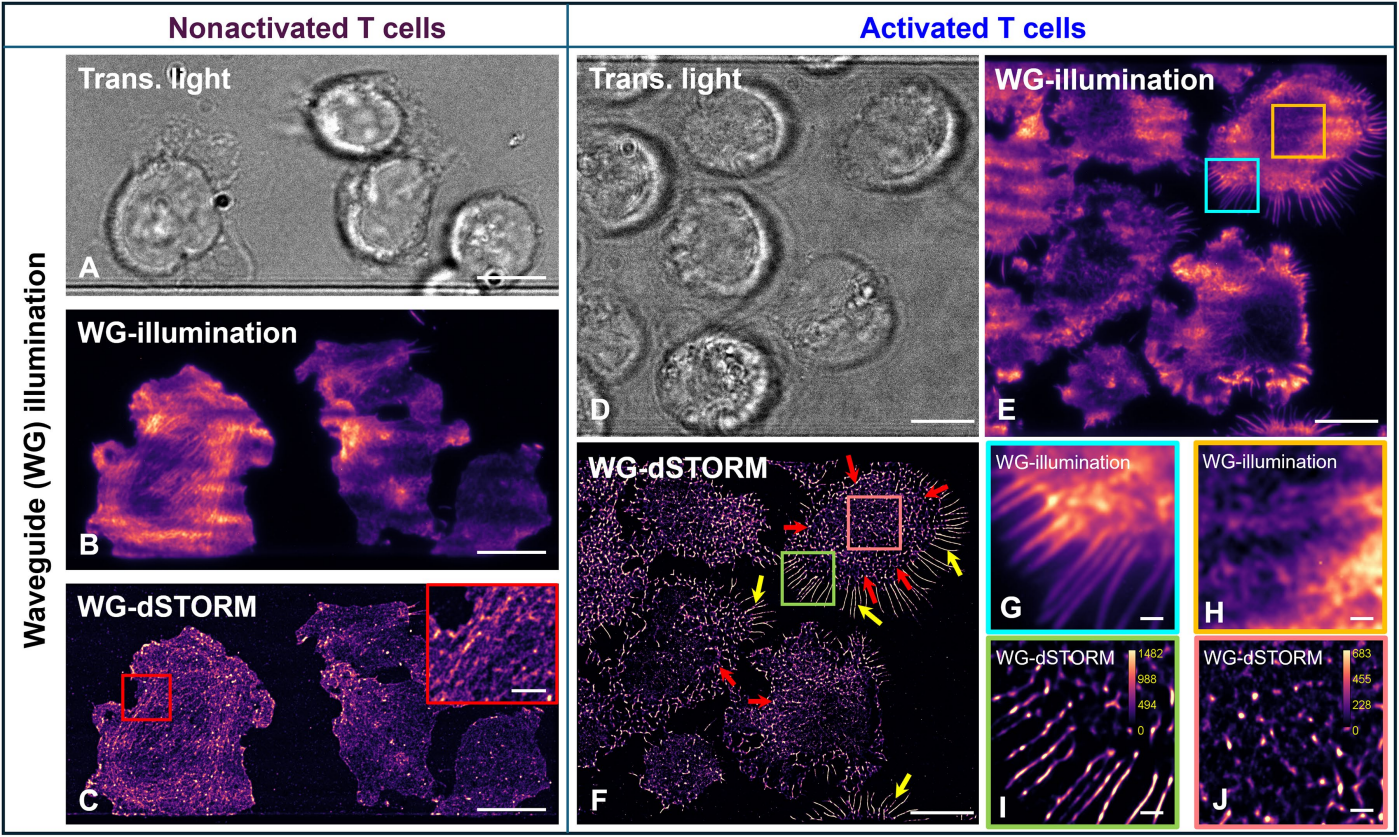
WG-dSTORM images of actin cytoskeleton rearrangements in Jurkat T cells on transparent polymer waveguide surfaces. The cells shown in the left-hand column are images of nonactivated T cells (cells are fixed on top of the nonfunctionalized, FN-coated waveguide surface), and the cells shown in the two right-hand columns are images of activated T cells (cells are fixed on top of the antibody-coated functionalized waveguide surface). (**A** and **B**) Trans. light and WG-illumination images of filamentous actin structures in nonactivated T cells, respectively. (**C**) Corresponding WG-dSTORM image of the actin network in the same T cells. The inset shows the enlarged view of the region of interest, marked with a red rectangular box. (**D** and **E**) Trans. light and WG-illumination images of filamentous actin structures in activated Jurkat T cells fixed on top of the antibody-coated functionalized waveguide surface, respectively. [The full FOVs of the transmitted light image and waveguide-illumination image (D and E) are shown in fig. S1 in the Supplementary Materials.] (**F**) Corresponding WG-dSTORM image of cortical actin networks of the same T cells. (**G** to **J**) Representative enlarged views of the regions of interest in WG-illumination wide-field (marked in cyan and orange) (G and H) and WG-dSTORM images (marked in light green and rose) (I and J) of actin filaments in activated T cells. Actin cytoskeleton rearrangements were observed in T cells following contact with the activating antibody-coated waveguide surface. The filaments are well resolved in WG-dSTORM images recorded for both nonactivated and activated T cells. Actin filaments are stained with Alexa Fluor 647 Phalloidin. Scale bars, 5 μm (A to F) and 1 μm (inset of C and G to J).

Waveguide TIRF excitation results in illumination of a thin-bottom section of the cells via an EF extending ~150 to 200 nm into the cells attached to the waveguide surface. The emitted fluorescence signal is collected through the transparent waveguide with a high-NA objective lens (see Materials and Methods). This reduces the detection of background signals resulting from scattering, refraction, and out-of-focus light during the imaging compared to the upright configuration. Figure 4 shows the waveguide TIRF illumination–based dSTORM (WG-dSTORM) images of actin filaments in Jurkat T cells seeded on top of the transparent photonic waveguide chips (the width of each waveguide is 60 μm). Figure 4 (A to C) shows the Trans. light, WG-illumination, and corresponding WG-dSTORM images of actin filaments in nonactivated T cells. An enlarged view of the ROI marked with a red box is shown in the inset of Fig. 4C. The WG-dSTORM image furthermore reveals that the actin fiber distribution in the nonactivated T cells is purely isotropic and that this actin network does not have the ability to function as a transportation network. In contrast, the WG-dSTORM images of actin filaments in activated Jurkat T cells [see Fig. 4 (D to F)] demonstrate that following the contact with the antibody-activated waveguide surface, Jurkat T cells become polarized, spread across the surface, and form an outward-growing rosette-shaped dense cortical actin network. Here, it is apparent that the distinct lamellipodial network formed by densely organized short actin filaments is overlaid by long actin filaments [see Fig. 4 (I and J)]. A faint contracted actin ring (marked by red arrows) and actin spikes (marked by yellow arrows) remain at the lamellipodium of the activated T cells. Furthermore, the WG-dSTORM images exhibit an inward-growing ramified actin network at the center of the interface, mostly formed at the later stage of activation. These are largely connected to the lamellipodial network within a few nanometers above the surface contact of activated T cells. This is further confirmed by the localization density calculations from two different regions of ramified and lamellipodial networks [calibration bars of Fig. 4 (I and J)]. It shows that the lamellipodial network (Fig. 4I) exhibits higher localization density compared to the ramified network (Fig. 4J). This seems reasonable as the lamellipodial actin network attaches in substantially closer proximity to the waveguide surface than the ramified actin network and is therefore exposed to a higher intensity of the EF, resulting in a higher localization density. These results also suggest that in activated T cells, the ramified and lamellipodial networks are contracted, and substantially denser actin networks are observed at the interface. Furthermore, individual filaments can be resolved from the highly dense cortical actin networks using waveguide TIRF illumination–based superresolution microscopy.

**Fig. 5.**
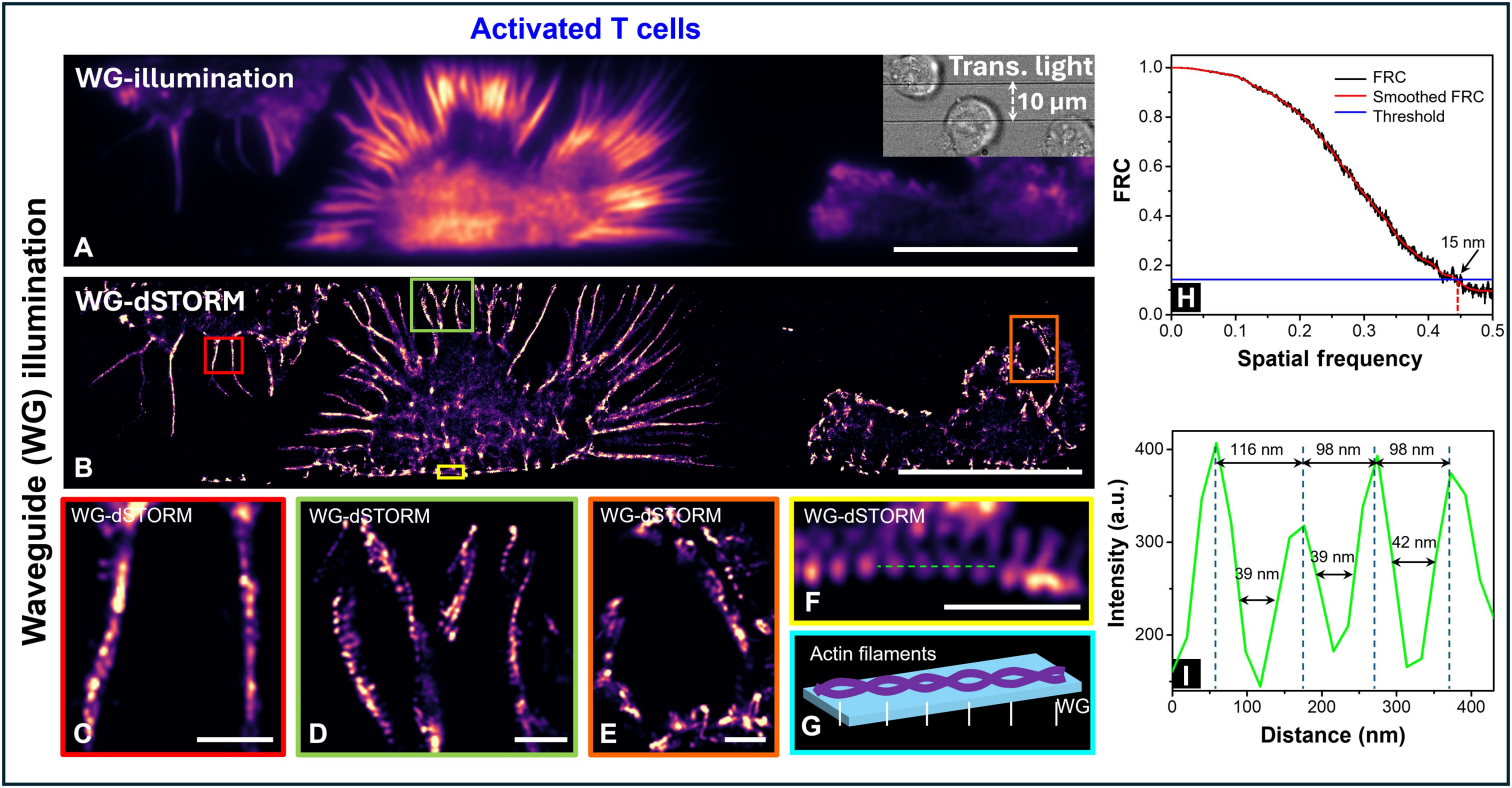
Nanoscopic details of actin cytoskeleton rearrangements in activated Jurkat T cells on a narrow-width waveguide surface with a width of 10 μm. (**A**) Diffraction-limited WG-illumination fluorescence image of the actin cytoskeleton in antibody-activated T cells on a 10-μm-wide polymer waveguide surface. The inset shows the corresponding Trans. light image. (**B**) WG-dSTORM image of the corresponding T cells over the same FOV. (**C** to **F**) Representative enlarged views of regions of interest in WG-dSTORM images (marked with red, light green, orange, and yellow boxes) of actin filaments in activated T cells. The narrow-width waveguide provides rather high illumination intensities, which result in higher EF strengths. WG-dSTORM provides sufficient spatial resolution (~15 nm) to observe the cytoskeleton structures of individual actin filaments in activated T cells at the IS. (**G**) Schematic representation of the actin filaments attached to the waveguide surface and the crossover of individual actin filaments through twisting and bending. Actin filaments are stained with Alexa Fluor 647 Phalloidin. (**H**) FRC calculation for Fig. 7B. (**I**) Line profile of Fig. 7F (dashed green line). a.u., arbitrary units. Scale bars, 10 μm (A and B) and 500 nm (C to F).

To further analyze the ultrastructure of actin filaments formed during T cell activation, we acquired dSTORM images using narrow-width waveguides for TIRF illumination. By using only 10-μm-wide waveguides instead of the 60-μm-wide waveguides, the higher EF intensity allows us to resolve individual actin filaments with higher localization precision. In Fig. 5 (A, inset of A, and B), the WG-illumination, Trans. light, and corresponding reconstructed WG-dSTORM images of actin filaments in activated T cells on top of a 10-μm-wide polymer waveguide are shown. The dSTORM images here resolve an apparent double-stranded helical structure of actin filaments [see Fig. 5 (C to F)]. The measured average crossover length obtained from these dSTORM images is ~40 nm (Fig. 5I). A similar double-stranded helical structure of actin filaments, where the two intertwined strands of monomeric chain of actin filament cross over each other by twisting and bending, was previously resolved by EM. Using EM, the crossover length was determined to be ~37 nm (*45*, *46*). The overall resolution enhancement in average crossover length of isolated actin filaments dSTORM images was determined by FRC analysis and measured to be ~15 nm (Fig. 5H). A total of 30,000 individual frames were used to reconstruct the dSTORM images across the entire FOV.

To place all these observations in perspective, we would like to note that compared with conventional objective-type TIRF nanoscopy and other superresolution approaches, the transparent waveguide platform offers a useful combination of large-area EF excitation, flexible high-NA fluorescence collection, and multimodal imaging compatibility on the same sample. Further results show that waveguide TIRF-dSTORM provides improved lateral resolution compared to objective-type TIRF-dSTORM at the waveguide surface, because the local excitation intensity of EFs in waveguide TIRF excitation is higher than the objective-TIRF excitation and the EF strength decays faster with distance if the index of refraction is higher and the decay length is shorter, as shown in fig. S3 (Supplementary Materials). In the present work, this allowed us to use WG-TIRF–based dSTORM imaging to distinguish dense peripheral lamellipodial actin networks from the more central ramified networks over a larger area than accessible with objective-type TIRF imaging. At the same time, dSTORM also provides a higher spatial resolution than what can typically be achieved by other superresolution microscopies used in previous T cell IS studies, i.e., STED and SIM. We found that for a given excitation laser power, only the narrow-width waveguides used in the present study generate sufficiently high EF strengths to allow us to visualize actin features with crossover spacing consistent with previous EM observations by dSTORM. At the same time, the present implementation is limited to cells fixed to a transparent surface, and it involves a trade-off between the FOV and the excitation intensity depending on the waveguide width; it should therefore be viewed as complementary to established live-cell and 3D superresolution methods providing lower spatial resolution.

In summary, this study demonstrates the broad capabilities that photonic waveguide chips provide for TIRF-based imaging of cortical actin networks in Jurkat T cells. Transparent polymer waveguides of varying width enable imaging across a large FOV with very high spatial resolution. We used these versatile platforms to investigate the organization and rearrangement of actin filaments in both antibody-activated and nonactivated fixed Jurkat T cells. WG-dSTORM images resolve individual filaments of the very dense cortical actin network with subdiffraction limit spatial resolution. Our results confirm the formation of outward-growing rings composed of a lamellipodial actin network during activation. In the later stage of activation, the formation of inward-growing ramified actin networks was observed in antibody-activated T cells. These constructs are involved in IS formation and are accompanied by actin reorganization and global cytoskeleton rearrangements. Furthermore, WG-dSTORM imaging resolves the double-stranded helical structure of actin filaments where two chains of monomeric actin cross over each other by twisting and bending. dSTORM images recorded using narrow-width waveguides (10-μm-wide waveguide) revealed that the average crossover length of actin filaments in T cells is ~40 nm. This suggests that the narrow-width waveguides can provide the rather high illumination intensities that are typically required for high-resolution dSTORM imaging. Thus, photonic waveguide chip–based single-molecule localization imaging, specifically the WG-dSTORM approach, provides an excellent platform for complementary superresolution imaging and presents an important step toward studying the cytoskeletal actin network within T cell ISs. Further results suggest that waveguide chip–based TIRF microscopy has the potential to surpass other traditional ways of superresolution imaging of intercellular cytoskeleton reorganization in terms of ease of use, compatibility with existing microscopes, and spatial resolution.

## MATERIALS AND METHODS

### Cell culture

Jurkat T cells (DSMZ no. ACC 282) were cultured in sterile RPMI (StableCell RPMI-1640 medium, R2405, Sigma-Aldrich) supplemented with 10% fetal bovine serum (PAA), 2 mM l-glutamine (Sigma-Aldrich), 1 mM sodium pyruvate (Sigma-Aldrich), 10 mM Hepes (Sigma-Aldrich), and 1% penicillin-streptomycin-neomycin solution (Sigma-Aldrich) using a standard protocol (*3*). Briefly, a humidified air environment was maintained at 37°C and 5% CO_2_ (carbon dioxide) during the cell culture, and handling was performed inside the HEPA (high-efficiency particulate air)–filtered microbiological safety cabinets. Cells were resuspended in fresh media and split every 2 days to ensure that the cells were in the logarithmic growth phase. The cell density was between 5 × 10^5^ and 9 × 10^5^ cells/ml.

### Microscope coverslip preparation

To visualize the cytoskeletal actin structure and conjugated cells in a horizontal direction with superresolution imaging, the Jurkat T cells were seeded on top of standard microscope coverslips (#1.5 high precision, 22 by 22 mm; cat. no. 0107052; Marienfeld GmbH, Germany; thickness, 170 ± 5 μm). At first, the cleaned coverslips were coated with 0.1% (w/v) PLL solution (Sigma-Aldrich, P8920) at room temperature for 15 min to allow the T cells to attach to the coverslip surface more adhesively. The PLL solution was then removed, and the coverslips were washed three times with phosphate-buffered saline (PBS; Sigma-Aldrich, D8662) and dried at 37°C for 1 hour.

### Sample preparation and dSTORM imaging (T cells on PLL-coated coverslips)

**Fig. 6.**
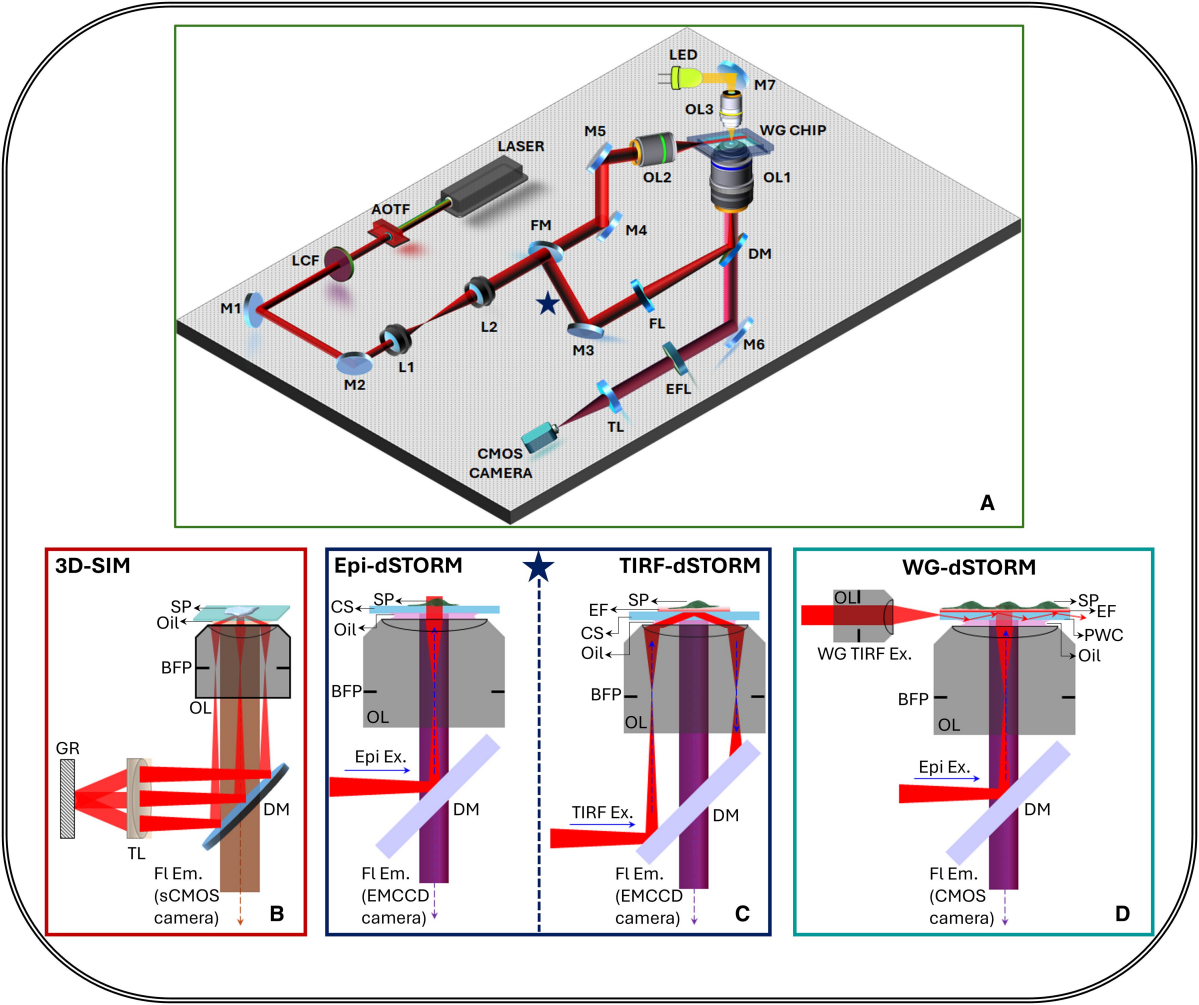
Schematic representation of the custom-built photonic waveguide chip–based nanoscopy setup and three different optical nanoscopy modalities. (**A**) Custom-built experimental setup. Laser lines were selected using an acousto-optic tunable filter, passed through a narrow bandpass laser cleanup filter, and subsequently expanded using a beam expander. A flip mirror was used to interchange the Epi and waveguide TIRF excitations. Epi excitation and fluorescence emission of the sample are collected by an objective lens (OL1) through the transparent substrate. A second objective lens (OL2) mounted on an *xyz* piezo stage is used to facilitate easy coupling of the excitation beam into the waveguide. The bright-field imaging unit of the setup consists of a white light light-emitting diode for illumination, a beam splitter, an objective lens (OL3), and a camera. It allows us to inspect the input facet of waveguide during laser beam coupling. (**B**) Schematic overview of the 3D-SIM setup. A sinusoidal illumination pattern is created by the interference of three laser beams in the sample plane. Cells are illuminated with this striped excitation pattern. The fluorescence signal is detected by the same objective lens and transmitted through a dichroic mirror to the camera. (**C**) Schematic representation of the Epi and objective-type TIRF illumination–based dSTORM imaging (“Epi-dSTORM” and “TIRF-dSTORM”), respectively. The corresponding excitation and emission geometries are modified in the experimental setup (marked by a blue star). (**D**) Schematic of WG-dSTORM. AOTF, acousto-optic tunable filter; LCF, laser cleanup filter; M, mirror; L, lens; FM, flip mirror; FL, focusing lens; DM, dichroic mirror; OL, objective lens; WG chip, waveguide chip; EFL, emission filter; TL, tube lens; SP, specimen; BFP, back focal plane; GR, grating; CS, coverslip; Ex., excitation; Fl Em., fluorescence emission; PWC, photonic waveguide chip.

For single-molecule localization microscopy, in particular dSTORM (*43*) imaging of actin filaments, Jurkat T cells were washed and suspended with cytoskeleton buffer [50 mM imidazole, 50 mM KCl, 0.5 mM MgCl_2_, 0.1 mM EDTA, and 1 mM EGTA (pH 6.8)] and incubated for 30 min at room temperature. Subsequently, the cells were fixed with the fixation buffer [paraformaldehyde (16%, w/v), methanol-free; 043368.9M, Thermo Scientific Chemicals) and a 1× PBS solution for 2 hours at 4°C. The fixative was removed, and the cells were washed twice with PBS by centrifugation. Cells were then incubated for 30 min in a permeabilization buffer (0.01% Triton X-100 solution; BP151-100, Thermo Fisher Scientific) to break down the cell membranes. Cells were washed twice with PBS by centrifugation. An aliquot of the washed cell solution in PBS was then placed on the PLL-coated coverslip surface for 10 min, and the cells were allowed to adhere to the coverslip surface, followed by three times washing with PBS. Cells were then incubated with Alexa Fluor 647 Phalloidin (1 unit/ml, 1:40; Alexa Fluor 647 Phalloidin, far-red, 650/668 nm; cat. no A22287; Invitrogen Phalloidin Labeling Probes) in a 1× PBS solution for 30 min at room temperature. Last, the sample was washed three times with a PBS solution, and dSTORM imaging buffer was added before imaging. The imaging buffer was prepared by using the common GODCAT buffer system (*43*), containing enzymatic oxygen scavengers (Sigma-Aldrich), glucose oxidase (Sigma-Aldrich), and catalase with β-mercaptoethylamine (MEA) as a switching agent (cysteamine; Sigma-Aldrich). The stock solutions of enzymes, glucose, and MEA (1 M) were prepared according to van de Linde *et al.* (*43*) and stored at −20°C until they were used for dSTORM measurements. The cells mixed with freshly prepared dSTORM imaging buffer (50 mM MEA blinking buffer, pH 7.4) were immediately covered with glass coverslips to minimize their contact with oxygen. Hence, the Epi-dSTORM and TIRF-dSTORM imaging of actin filaments was performed using a custom-built wide-field inverted dSTORM microscopy setup with an objective-type TIRF configuration (Fig. 6, A and C). The details of the setup were described and reported previously (*39*, *40*, *42*). Briefly, dSTORM imaging was performed under continuous laser illumination of 647 nm (~1 kW/cm^2^). A 60×, 1.49-NA oil immersion TIRF objective lens (OLYMPUS Apo N, APON60XOTIRF) was used with an additional 2.5× magnification to collect fluorescence onto an EMCCD (electron-multiplying charge-coupled device) camera (iXon, X-1659, Andor), yielding a pixel size of 113 nm. A total of 20,000 frames with a camera exposure time of 40 ms were acquired (for Fig. 1) for dSTORM measurements, and superresolution SMLM images were reconstructed using the open-source ImageJ plug-in ThunderSTORM (*47*). Both the Epi-dSTORM and TIRF-dSTORM measurements distinguish individual actin filaments with cross-sectional widths well below the diffraction limit. However, we find that only the edges of activated T cells are attached to the surface and most of the remaining parts are not attached to the coverslip substrate properly. Therefore, to visualize the actin cytoskeleton structure in Jurkat T cells at the IS with high throughput and high resolution, the cells were seeded on top of the transparent polymer photonic waveguide chips for further experiments and measurements.

### Waveguide chip fabrication

**Fig. 7.**
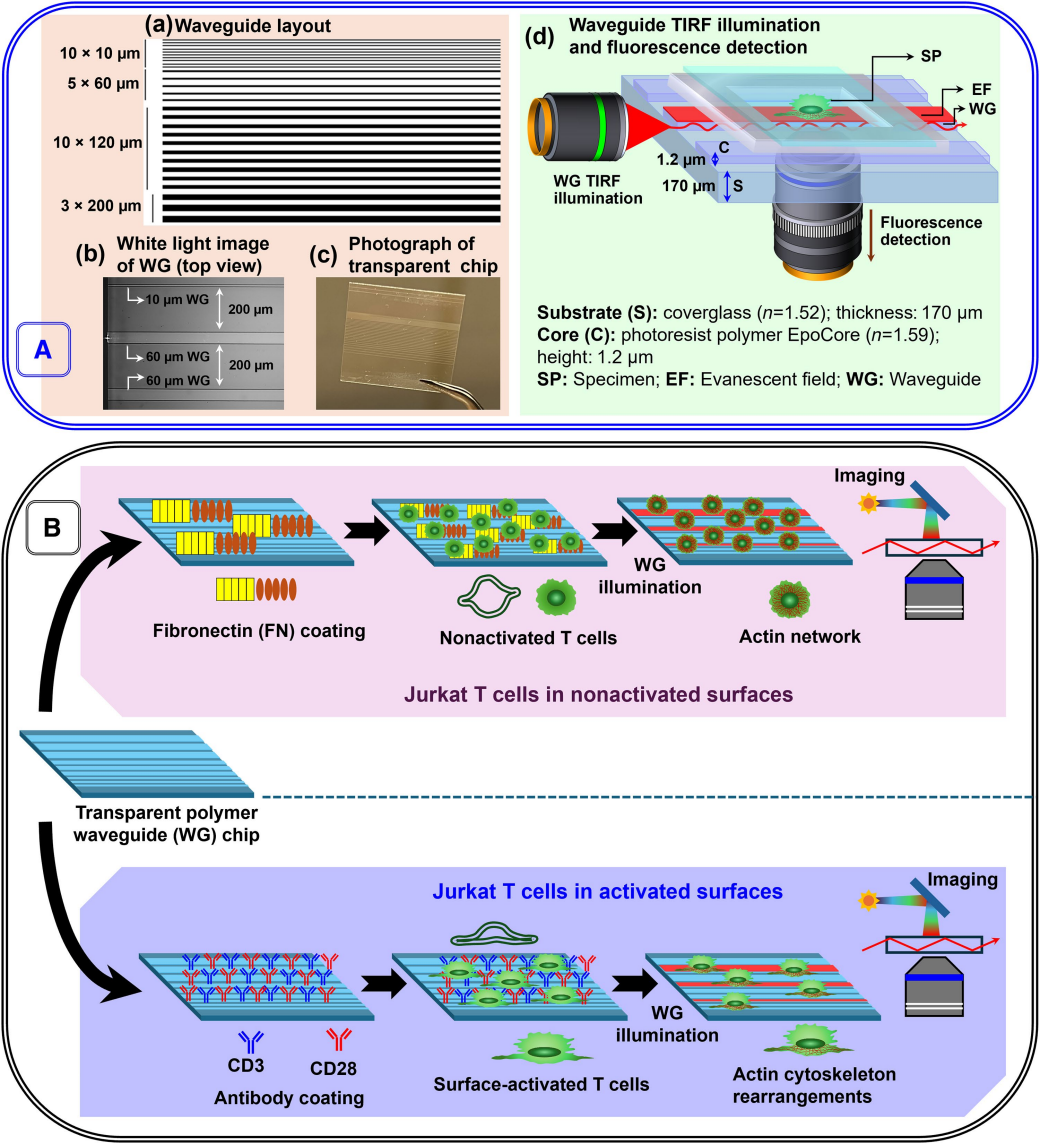
Schematic representation of the micrograph of waveguide chip layout, waveguide architecture, waveguide TIRF illumination, and detection and treatment of transparent polymer photonic waveguide chips, permitting the imaging of nonactivated (resting) T cells and activated T cells. (**A**) Micrograph of photonic waveguide chip and waveguide TIRF illumination. Details of the waveguide layout (a), white light image (top view) (b), photograph of a transparent photonic waveguide chip (c), and schematic of the waveguide TIRF illumination and fluorescence detection (d). The white light image (b) shows the top view of the actual architecture of 10- and 60-μm waveguides and the distance between them (200 μm). The photograph shows the transparent photonic chip. To the right, the schematics of the waveguide TIRF illumination and fluorescence detection are shown (d). Transparent planar polymer waveguides [core (C): EpoCore with refractive index, *n* = 1.59 for λ = 647 nm] with a thickness of 1.2 μm were fabricated on top of standard #1.5 borosilicate microscope coverslips [substrate (S): thickness, 170 μm]. (**B**) Schematic representation of the treatment of transparent polymer photonic waveguide chips, permitting the imaging of nonactivated (resting) T cells and activated T cells. For nonactivated T cells, the waveguide surface was coated with FN to allow the cells to correctly adhere to the surface. For T cell activation, the waveguide surface was first coated with CD3 and CD28 antibodies. Then, Jurkat T cells were seeded on top of the antibody-coated surface to activate the T cells and initiate the remodeling of the cortical actin cytoskeleton. Last, the actin structures were recorded via epi-excited as well as waveguide TIRF–excited fluorescence imaging.

The transparent waveguides were fabricated on top of a standard #1.5 borosilicate microscope coverslip (thickness: 170 μm). The photoresist polymer EpoCore (refractive index *n* = 1.59 for λ = 647 nm) was used as the core waveguide material and spin-cast on top of the glass substrate. The detailed procedure of the photonic chip fabrication process was reported previously (*39*, *40*, *42*). The schematic and micrographs of the waveguide chip layout, the waveguide architecture (top-view white light image and photograph of transparent chip), and the schematics of waveguide TIRF illumination and detection are shown in Fig. 7A. Briefly, a single photonic chip consists of several waveguides with different widths that are photolithographically produced on the coverslip substrate. The waveguides vary in width from 10 to 200 μm, and they are separated from each other by a distance of 200 μm. A group of 10 waveguides with a width of 10 μm each is prepared at the upper end of the chip, followed by a group of five waveguides with a width of 60 μm each. This is followed by a group of 10 waveguides with a width of 120 μm each, and a last group of three waveguides with a width of 200 μm each (Fig. 7A). The mean height of the fabricated waveguides is ~1.2 μm. The waveguide chips were cleaned by an ultrasonication bath using 0.1% SDS (436143, Sigma-Aldrich) water solution for 5 min. Then, the chips were rinsed three times with reagent grade ethanol followed by distilled water. The washed surface was dried in a stream of pressurized nitrogen gas flow. After that, a transparent polydimethylsiloxane chamber with a volume of ~200 μl was added on top of the waveguide chip to place the cell suspension inside it. Last, the waveguide chips were stored in sterilized petri dishes for further use.

### Sample preparation and T cell activation on waveguide chips

To visualize the organization and global rearrangements of actin networks with nanometer-scale localization precision, the actin cytoskeleton structure of both nonactivated (or resting) and surface-activated Jurkat T cells was imaged. To accomplish this, the waveguide chip surfaces were modified or functionalized differently, as shown in Fig. 7B. For nonactivated (or resting) T cells, the cleaned waveguide chips were coated with FN (0.2 mg ml^−1^ in PBS; F1141, Sigma-Aldrich, Bovine Fibronectin) for 45 min at room temperature and washed twice with PBS. To enable T cell activation, the waveguide chip surfaces were coated with IgG and anti-CD3 and anti-CD28 antibodies, following similar protocols as published (*3*). Briefly, waveguide chips were coated with 1 ml of Goat Anti-Mouse IgG antibody (50 μg/ml; Anti-Mouse IgG, M8642, Sigma-Aldrich) in coating buffer (50 mM Na_2_CO_3_ and 50 mM NaHCO_3_, pH 9.6) overnight at 4°C temperature. Waveguide chips were washed three times with a 1× PBS solution and blocked for 3 hours at room temperature using RPMI + 10% fetal bovine serum followed by three times washing with PBS. The washed surfaces were coated with 1:1 Anti-Human CD3 (14-0037-82, Invitrogen) and Anti-Human CD28 (14-0289-82, Invitrogen) at 5 μg/ml each in a PBS solution overnight at 4°C temperature. Last, the waveguide chips were washed three times with a PBS solution before seeding the cells.

### T cell activation, cell fixation, and labeling of actin filaments

Jurkat T cells were washed and suspended with the cytoskeleton buffer [50 mM imidazole, 50 mM KCl, 0.5 mM MgCl_2_, 0.1 mM EDTA, and 1 mM EGTA (pH 6.8)] and incubated for 30 min at room temperature. Two hundred microliters of cells was added dropwise evenly across the FN-coated and antibody-activated waveguide surfaces separately. Cells were incubated for 30 min in the incubator (at 37°C and 5% CO_2_). Subsequently, the cytoskeleton buffer was replaced, and the cells were fixed with the fixation buffer [4% (w/v) paraformaldehyde] and a 1× PBS solution for 2 hours at 4°C. The fixative was washed thrice with a PBS solution without disturbing the fixed cells attached to the waveguide chip surface. Cells were then incubated for 30 min in a permeabilization buffer (0.01% Triton X-100 solution) to break down the cell membranes followed by three times washing with PBS. Last, cells were incubated with Alexa Fluor 647 Phalloidin (1 unit/ml, 1:40 diluted) in a 1× PBS solution for 30 min at room temperature and washed three times with a PBS solution before adding the imaging buffer.

### 3D-SIM imaging of actin filaments in Jurkat T cells

To analyze the actin cytoskeletal structure or its rearrangement in antibody-activated T cells with respect to their nonactivated (or resting) counterparts, 3D-SIM images were acquired using a commercial SIM microscope (Deltavision OMXv4.0 BLAZE, GE Healthcare) equipped with a 60×, 1.42-NA objective lens (Fig. 6B). The details of SIM data acquisition and SIM image reconstructions were described in our previous publication (*39*). In short, this system is in the inverted microscope configuration, which means that the illumination light must penetrate through the glass coverslip (170-μm thickness) and the additional 1.2-μm-thick polymer waveguide to excite the cells attached to the waveguide surface. A red diode laser (λ = 642 nm) was used to excite fluorescence in the samples. We captured 1.5-μm-thick *z*-stacks with a step size of 0.125 μm and 15 raw images per slice (three angles with five phases each) for each cell. The raw images were reconstructed using the SoftWorX package from GE Healthcare to obtain the final 3D-SIM images. The FOV of the SIM reconstruction is limited to 40.96 μm by 40.96 μm. Before SIM measurements, a stable and nonfluctuating buffer system, which contains reducing and oxidizing agents (ROXS) (*48*) without thiols, was added to the sample to minimize the photobleaching of the sample during the measurements. After the completion of the SIM measurements, the ROXS buffer solution was removed, and the sample was washed with PBS before adding the dSTORM imaging buffer.

### Objective-type TIRF-dSTORM imaging of actin filaments in activated T cells

For objective-type TIRF-dSTORM measurements, the custom-built microscopy setup detailed in the “Sample preparation and dSTORM imaging (T cells on PLL-coated coverslips)” section (Fig. 6C) was used. To explore the rearrangement of the actin cytoskeleton in surface-activated T cells with high resolution, we first recorded epi-illumination and objective-type TIRF illumination dSTORM (Epi-dSTORM and TIRF-dSTORM, respectively) images of the cells. For this, the same TIRF objective lens (Apo N, APON60XOTIRF, Olympus) was used for both excitation and detection, and the fluorescence emission was recorded with an EMCCD camera (iXon, X-1659, Andor), as mentioned earlier (Fig. 6C). A total of 30,000 (for Epi) and 10,000 (for TIRF) frames with a camera exposure time of 40 ms each were acquired for dSTORM measurements, and superresolution SMLM images were reconstructed using the open-source ImageJ plug-in ThunderSTORM (*47*).

### Waveguide TIRF excitation dSTORM imaging (WG-dSTORM)

For WG-dSTORM measurements, a separate part (Fig. 6D) of the custom-built setup was used as shown in Fig. 6. The details of the setup and the procedure of coupling laser light into the waveguide chips were also reported previously (*39*). Briefly, an Ar-Kr+ ion laser (Innova 70C Spectrum, Coherent) was used as the laser source. The laser line at a 647-nm wavelength with a ∼150-mW laser power was selected using an acousto-optic tunable filter. The output laser beam was directed to the coupling optics on top of a Thorlabs NanoMax 300 *xyz* piezo stage (MAX312D, Thorlabs). A long-working-distance 20×, 0.35-NA objective lens (Olympus, SLMPlan, 20×/0.35, ∞/0) was used to focus the beam onto the input facet of the polymer waveguide. Focus spot optimizations for WG-TIRF illumination and minimization of interference streak patterns were done by controlling the *xyz* piezo stage position. A separate 60×, 1.35-NA oil immersion objective lens (UPlanSApo, Olympus) was used to collect fluorescence emissions. The dSTORM raw image stacks were acquired with an uncooled industry-grade CMOS (complementary metal-oxide semiconductor) camera (UI-3060CP-MGL, IDS Imaging Development Systems) with a 1936 by 1216–pixel sensor and a pixel size of 5.86 μm by 5.86 μm. The upright microscope was used to investigate the waveguide input facets during the measurement, as shown in Fig. 6A. The transparent nature of the polymer waveguide chip allows us to switch between WG-based TIRF illumination and epi-illumination on the same microscope setup. Cells mixed with freshly prepared dSTORM imaging buffer (GODCAT buffer) were immediately covered with a glass coverslip to minimize the contact with oxygen. For localization microscopy on polymer waveguide chips (WG-dSTORM), the excitation power at the front facet was ~20 mW for the 647-nm laser line. The acquisition time was 50 ms per frame, and a total of 50,000 frames (for nonactivated T cells, Fig. 4C) and 35,000 frames (for activated T cells, Fig. 4F) were recorded using the CMOS camera and used for dSTORM image reconstruction. The superresolution dSTORM images were reconstructed from the series of acquired raw images using the open-source comprehensive Superresolution Microscopy Analysis Platform (SMAP) (*49*). Last, the localization tables are imported in the ImageJ plug-in ThunderSTORM (*47*) and visualized as average shifted histograms.

### Naming conventions

Three different optical nanoscopy modalities with several excitation and emission geometries were used to image the actin cytoskeleton structure of Jurkat T cells and compared with their corresponding bright-field and diffraction-limited wide-field images. For bright-field images, the transmitted light was collected by using bright-field white light illumination and named as transmitted light (“Trans. light” images). For diffraction-limited wide-field images, epi-illumination (“Epi”) and objective-type TIRF illumination (“TIRF”) were used. dSTORM images recorded with Epi and objective-type TIRF illumination were further referred to as “Epi-dSTORM” and “TIRF-dSTORM,” respectively. Furthermore, the images recorded with waveguide TIRF excitation were referred to as “WG-illumination” for diffraction-limited wide-field images and “WG-dSTORM” for waveguide-illuminated dSTORM images.
