# Supplementary material for: Photonic waveguide chip–based nanoscopy visualizes rearrangements of the cortical actin cytoskeleton in activated Jurkat T cells
Source: Sci Adv. 2026 Jul 15;12(29):eaeg3960. doi: 10.1126/sciadv.aeg3960 (PMC13371924; doi:10.1126/sciadv.aeg3960)
Supplement: Supplementary file 1 — Figs. S1 to S3 [file sciadv.aeg3960_sm.pdf]

Science Advances
AAAS

Supplementary Materials for

Photonic waveguide chip–based nanoscopy visualizes rearrangements of the cortical actin cytoskeleton in activated Jurkat T cells

Surjendu Bikash Dutta *et al.*

Corresponding author: Thomas Huser, thomas.huser@physik.uni-bielefeld.de

Sci. Adv. **12**, eaeg3960 (2026)
DOI: 10.1126/sciadv.aeg3960

This PDF file includes:

Figs. S1 to S3

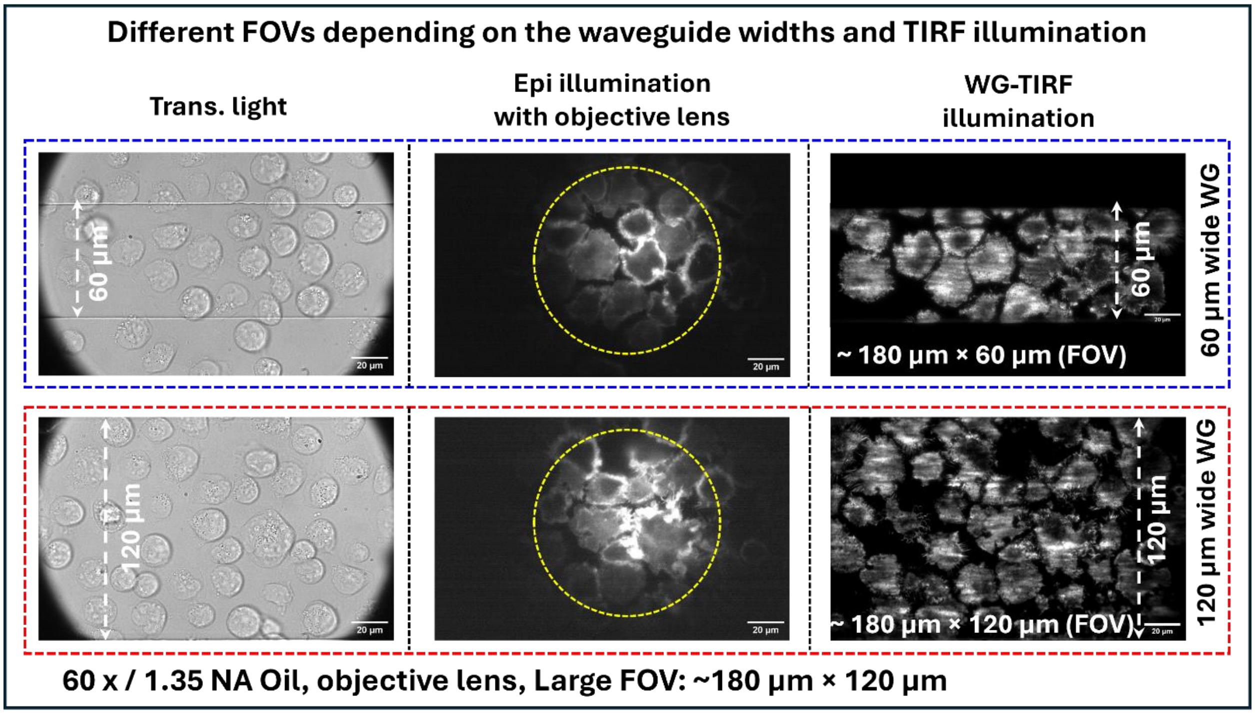

Fig. S1: *Measurements of the different fields of view (FOVs) depending on the waveguide widths and total internal reflection fluorescence (TIRF) illumination. The left most column presents the bright field images (Trans. light) of two different fields of view for 60 µm and 120 µm wide waveguides. A part of the transmitted light image as well as waveguide TIRF (WG-TIRF) illumination wide field image (upper panel of Fig. S1, surrounded by blue dashed line) of a 60 µm wide waveguide are also shown in detail in Figure 4 in the main manuscript. The middle column presents the diffraction limited wide field fluorescence images of actin cytoskeleton structures of surface activated Jurkat T cells attached to the waveguide surfaces, in this case the excitation is based on epi illumination with an objective lens (60x/ 1.35 NA, Oil immersion objective lens). Here, only the central regions, surrounded by yellow dashed lines, provide sufficient fluorescence signals - limiting the available FOV. The right-most column presents diffraction limited wide field fluorescence images of actin cytoskeleton networks of the same surface activated Jurkat T cells attached to the waveguide surfaces by WG-TIRF based illumination. In this modality an evanescent field is generated on top of the waveguide surface (where the waveguide has a higher index of refraction than its surroundings). This evanescent field can then be used for total internal reflection fluorescence excitation over an almost arbitrarily large field of view (FOV). This results*

in two different FOVs for a given detection objective lens, provided by two different widths of waveguides.

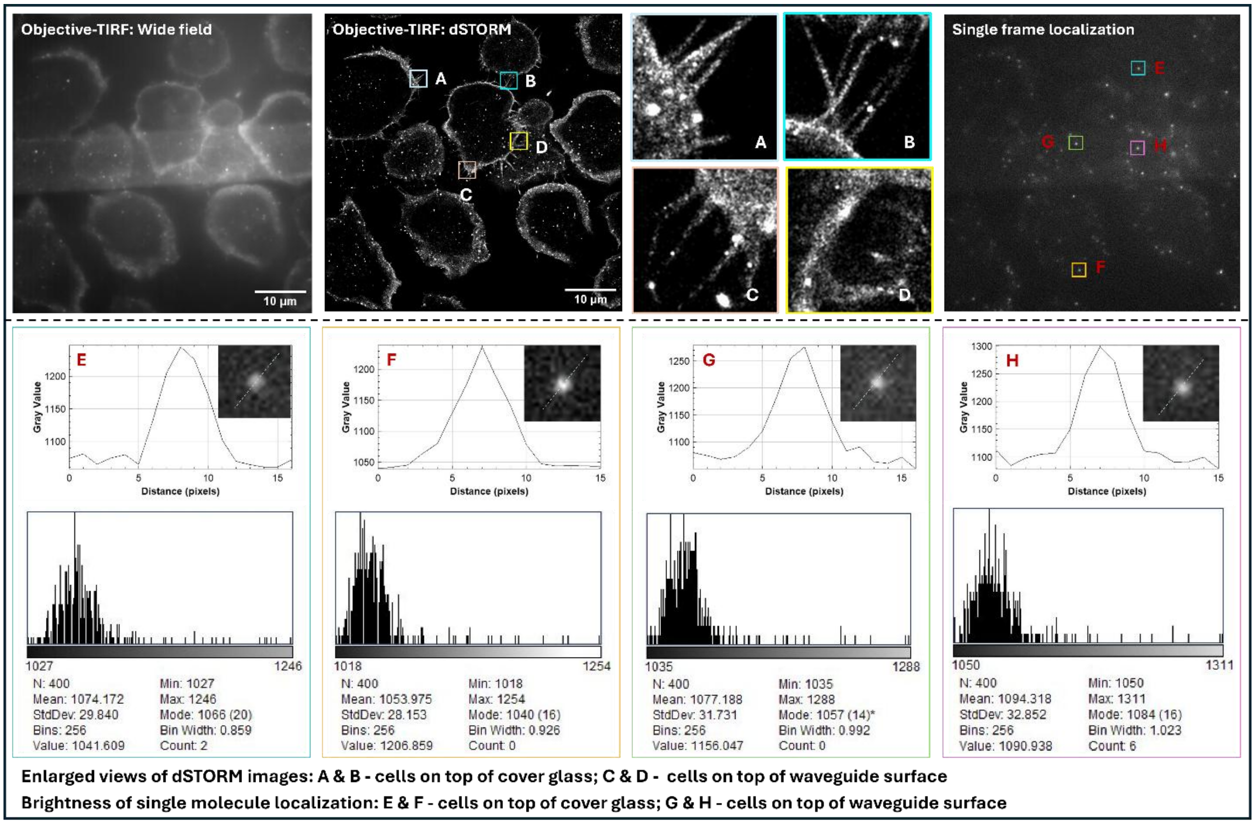

Fig. S2: *Single molecule brightness values recorded for cells sitting on a bare cover glass surface and on a polymer waveguide surface where both are excited by objective-type TIRF illumination. The top left corner image shows the diffraction-limited wide field images of the actin cytoskeleton of surface activated Jurkat T cells for a measured FOV (wide-field meaning that, in this case, no single molecule localization was used). In this FOV, some cells are sitting on top of the waveguide surface (10 µm wide waveguide), and some cells are sitting on the nearby cover glass substrate. The top middle part of the figure shows the reconstructed dSTORM images of the same FOV with different regions of interests,* ***A*** *&* ***B****) showing cells on top of the bare cover glass surface, and* ***C*** *&* ***D****) cells on top of the waveguide surface. The dSTORM measurement and reconstruction show no major differences for the different substrates. The top right corner image shows the measured raw single molecule localization data of a single frame of imaging. The lower part of the figure shows the brightness values of single molecules of the given single frame of localization data*

*recorded from the bare cover glass surface (**E** & **F**) and from the waveguide surface (**G** & **H**), respectively. This clearly shows that significant fluorescence events were detected in both cases, and no notable differences were observed. All the experimental parameters and conditions remained the same as well.*

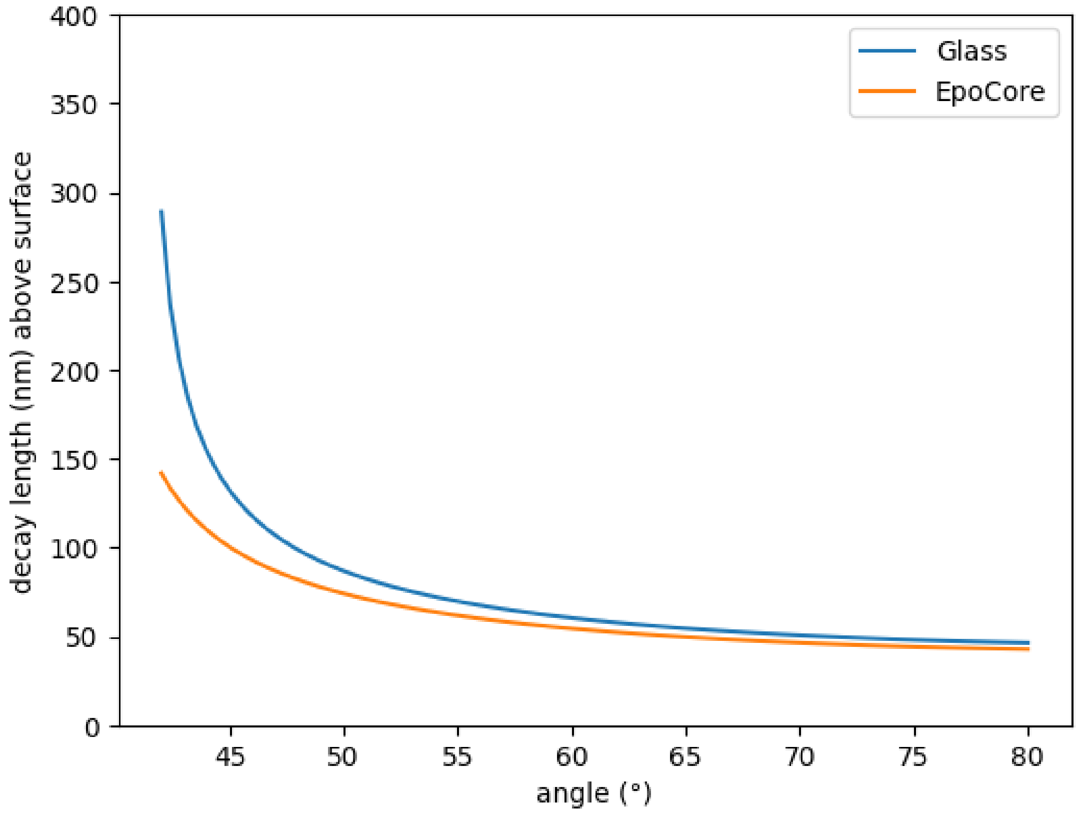

Fig. S3: *The simulated 1/e decay length (d) of the electric field in an aqueous solution under TIRF illumination for a glass substrate and an EpoCore substrate (the polymer waveguide material used in our experiments). The simulation shows that the electric field decays more strongly for all angles above the critical angle for the higher index material (EpoCore), leading to a higher electric field strength that is concentrated to the first few 10s of nanometers above the substrate. In the figure the decay length is plotted against the angle at which a laser beams hits the interface between the substrate and the aqueous solution. For objective-type TIRF, this angle is, in most experiments as well as in our case, usually close to the critical angle and rarely goes above 60°, as objectives with a higher numerical aperture (NA) are required to reach those more extreme angles. In the*

case of the EpoCore waveguide, different angles are reached for different modes supported within the waveguide and it is rather difficult to determine the precise conditions (angles) under which waveguide-TIRF illumination occurs.
